# Continuing Persistence and Biomagnification of DDT and Metabolites in Northern Temperate Fruit Orchard Avian Food Chains

**DOI:** 10.1002/etc.5220

**Published:** 2021-11-10

**Authors:** Robert Kesic, John E. Elliott, Kate M. Fremlin, Lewis Gauthier, Kenneth G. Drouillard, Christine A. Bishop

**Affiliations:** ^1^ Department of Biological Sciences Simon Fraser University Burnaby British Columbia Canada; ^2^ Environment and Climate Change Canada, Wildlife Research Division Delta British Columbia Canada; ^3^ Environment and Climate Change Canada, National Wildlife Research Centre Ottawa Ontario Canada; ^4^ Great Lakes Institute for Environmental Research University of Windsor Windsor Ontario Canada

**Keywords:** DDT, Earthworms, American robins, Food chain, Biomagnification, Fugacity

## Abstract

Dichlorodiphenyldichlorethane (1,1,1‐trichloro‐2,2‐bis(p‐chlorophenyl)ethane) (DDT) is an organochlorine insecticide that was widely used from the late 1940s to the 1970s in fruit orchards in the Okanagan valley, British Columbia, Canada, and in the process, contaminated American robin (*Turdus migratorius*) food chains with the parent compound and metabolite dichlorodiphenyldichloroethylene (1,1‐dichloro‐2,2‐bis(4‐chlorophenyl)ethylene) (*p,p*′‐DDE). In the present study, we examined the biological fate of these DDT‐related (DDT‐r) compounds at the same sites/region 26 years after a previous study by: (1) collecting soil, earthworms, and American robin eggs from apple, cherry, and pear orchards; (2) characterizing the diet and trophic positions of our biota using stable isotope analyses of δ^13^C and δ^15^N; and (3) estimating fugacity, biota‐soil‐accumulation factors (BSAFs), and biomagnification factors (BMFs). Mean *p,p*′‐DDE concentrations (soil: 16.1 µg/g organic carbon‐lipid equivalent; earthworms: 96.5 µg/g lipid equivalent; eggs: 568 µg/g lipid equivalent) revealed that contamination is present at elevated levels similar to the 1990s and our average soil DDE:DDT ratio of 1.42 confirmed that DDT is slowly degrading. American robins appeared to feed at similar trophic levels, but on different earthworms as indicated by egg stable isotope values (mean δ^15^N = 8.51‰ ± 0.25; δ^13^C = −26.32‰ ± 0.12). *Lumbricidae* and *Aporrectodea* worms shared a roughly similar δ^15^N value; however, *Lumbricus terrestris* showed a markedly enriched δ^13^C isotope, suggesting differences in organic matter consumption and physiological bioavailability. Biota‐soil‐accumulation factors and BMFs ranged over several orders of magnitude and were generally >1 and our fugacity analyses suggested that *p,p*′‐DDE is still thermodynamically biomagnifying in American robin food chains. Our results demonstrate that DDT‐r in fruit orchards remains bioavailable to free‐living terrestrial passerines and may pose a potential toxicological risk. *Environ Toxicol Chem* 2021;40:3379–3391. © 2021 Her Majesty the Queen in Right of Canada. *Environmental Toxicology and Chemistry* published by Wiley Periodicals LLC on behalf of SETAC. Reproduced with the permission of the Minister of Environment and Climate Change Canada.

## Graphical abstract

American robins nesting in apple, cherry, and pear fruit orchards in the Okanagan valley region of British Columbia, Canada are still being exposed to elevated dichlorodiphenyldichlorethane‐related concentrations via a soil‐earthworm‐egg food chain.

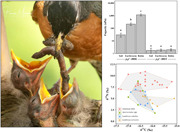

## INTRODUCTION

Although use of dichlorodiphenyldichlorethane (1,1,1‐trichloro‐2,2‐bis(p‐chlorophenyl)ethane) (*p,p*′‐DDT) has been highly restricted in North America since the 1970s, residues of *p,p*′‐DDT and its highly persistent metabolite, dichlorodiphenyldichloroethylene (1,1‐dichloro‐2,2‐bis(4‐chlorophenyl)ethylene) (*p,p*′‐DDE) continue to be detected in environmental media. The trophic transfer, or biomagnification, of these DDT‐related (DDT‐r) compounds in food webs has historically been associated with eggshell quality effects and population declines of raptors and piscivorous birds, which have been the focus of many contaminant monitoring programs and toxicological studies in the northern and southern hemispheres (Blus, 2011; Braune et al., 2018; Elliott & Martin, 1994; Elliott et al., 1988; 1996; 2005; 2021; Grier, 1983; Hickey & Anderson, 1968; Ratcliffe, 1967; Umulisa et al., 2020). However, over the past 40 years, elevated concentrations of *p,p*′‐DDE have been detected in fruit orchard agroecosystems, including terrestrial passerines and their eggs due to soil‐to‐invertebrate bioaccumulation pathways (Bishop et al., 2000; Blus et al., 1987; Elliottet al., 1994; Gill et al., 2003; Harris et al., 2000; Hebert et al., 1994). Exposure to environmentally relevant levels of *p,p*′‐DDE in fruit orchards may be associated with reduced egg/chick survival (Bishop et al., 2000), neuro‐system effects (Iwaniuk et al., 2006), and potential impaired reproduction of avian predators (Elliott et al., 2005).

The Okanagan valley is an intensive fruit growing region located in the south‐central interior of British Columbia, Canada, that was heavily treated with DDT to mitigate codling moth (*Cydia pomonella L*.) outbreaks and other insect pests (Kuo et al., 2012). Between 1946 and 1970, approximately 30 kg of *p,p*′‐DDT/ha/year could have been applied to fruit orchards, which is three times more than the recommended application rate in other Canadian provinces (Harris et al., 2000). The Okanagan valley was therefore one of the most heavily DDT sprayed agricultural regions in Canada. During the early 1990s, elevated levels of *p,p*′‐DDE were detected in the eggs of several songbird species nesting in Okanagan valley fruit orchards (Elliott et al., 1994). Of those species, American robins (*Turdus migratorius*) had *p,p*′‐DDE concentrations nearly 100 times higher than in the eggs of neotropical migrant species (tree swallow, *Tachycineta bicolor*; barn swallow, *Hirundo rustica*; house wren, *Troglodytes aedon*) and other year‐round non‐migratory residents (California quail, *Callipepla californica*; black‐billed magpie, *Pica pica*), suggesting that robins were acquiring *p,p*′‐DDE locally and not on their wintering grounds (Elliott et al., 1994). Several American robin food chain studies were conducted following the use of DDT for Dutch elm disease control (Barker, 1958; Beaver, 1980; Hunt, 1969b) and spruce budworm control (Dimond et al., 1970; Knupp et al., 1976); however, a food chain study conducted in the Okanagan valley by Harris et al. (2000) confirmed that American robins were bioaccumulating legacy DDT‐r through earthworm exposure, with *p,p′‐*DDE concentrations in robin eggs averaging 85 µg/g (wet wt) in orchards and 8.22 µg/g in non‐orchard areas. In a parallel study, American robin eggs collected from various Okanagan valley orchards between 1997 and 1998 contained up to 302 µg/g *p,p*′‐DDE; the highest reported concentration of any bird, mammal, or invertebrate assessed in the region (Gill et al., 2003).

Few assessments of the biomagnification potential of DDT‐r in American robins and their prey have been conducted, largely due to a high degree of inter‐ and intra‐orchard variability in DDT‐r contamination, insufficient biomass collection of earthworm communities, and not comparing DDT‐r concentrations in biota on a lipid normalized basis. Consequently, there is a need to re‐evaluate the bioaccumulation and biomagnification potential of DDT‐r in these terrestrial food chains, which may also be useful in ecological risk assessments. Temporal comparisons of field bioaccumulation, biomagnification, and biotransformation of DDT‐r have also been largely understudied, especially in the context of free‐living terrestrial passerines in which DDT‐r could still bioaccumulate and pose an ecotoxicological risk (Bishop et al., 2000). American robins provide a useful model to investigate the uptake and trophic transfer of DDT‐r because: (1) they are a common year‐round resident with a fairly heterogenous distribution in the Okanagan valley and readily nest in orchards (Cannings et al., 1987), allowing for within‐species comparisons across different spatial scales; (2) they forage near their nest sites and during the breeding season will feed almost exclusively on earthworms, which are known to bioaccumulate high DDT‐r levels (Harris et al., 2000), thereby providing a direct link of contamination from the soil; (3) they have a long nesting season and can produce up to three broods (Vanderhoff et al., 2020), facilitating a large sample size in a wild species; (4) their eggs can be used as a sampling matrix to represent contamination from the local area because DDT‐r and other lipophilic contaminants are mobilized and partitioned into eggs prior to and during the egg‐laying period (Elliott et al., 2021); and (5) they provide a useful indication of the ongoing DDT‐r exposure to other resident and migratory birds, including raptors (Brogan et al., 2017; Elliott et al., 2005) where *p,p*′‐DDE and other contaminants are potentially biomagnified to a greater extent.

The objectives of our study were to examine the biological fate of DDT‐r in a simplified terrestrial food chain 26 years after a previous study by: (1) collecting soil, earthworms, and American robin eggs from apple, cherry, and pear orchards with the prediction that DDT‐r and other halogenated contaminant concentrations would significantly decline; (2) evaluating differences in trophic relationships, foraging habitat, and dietary uptake of DDT‐r between different earthworm species and American robins using stable isotope analyses of δ^13^C and δ^15^N; and (3) estimating chemical fugacity, biota‐soil‐accumulation factors (BSAFs), and biomagnification factors (BMFs) on a lipid normalized basis.

## MATERIALS AND METHODS

### Sampling orchards and biota collection sites

**Figure 1:**
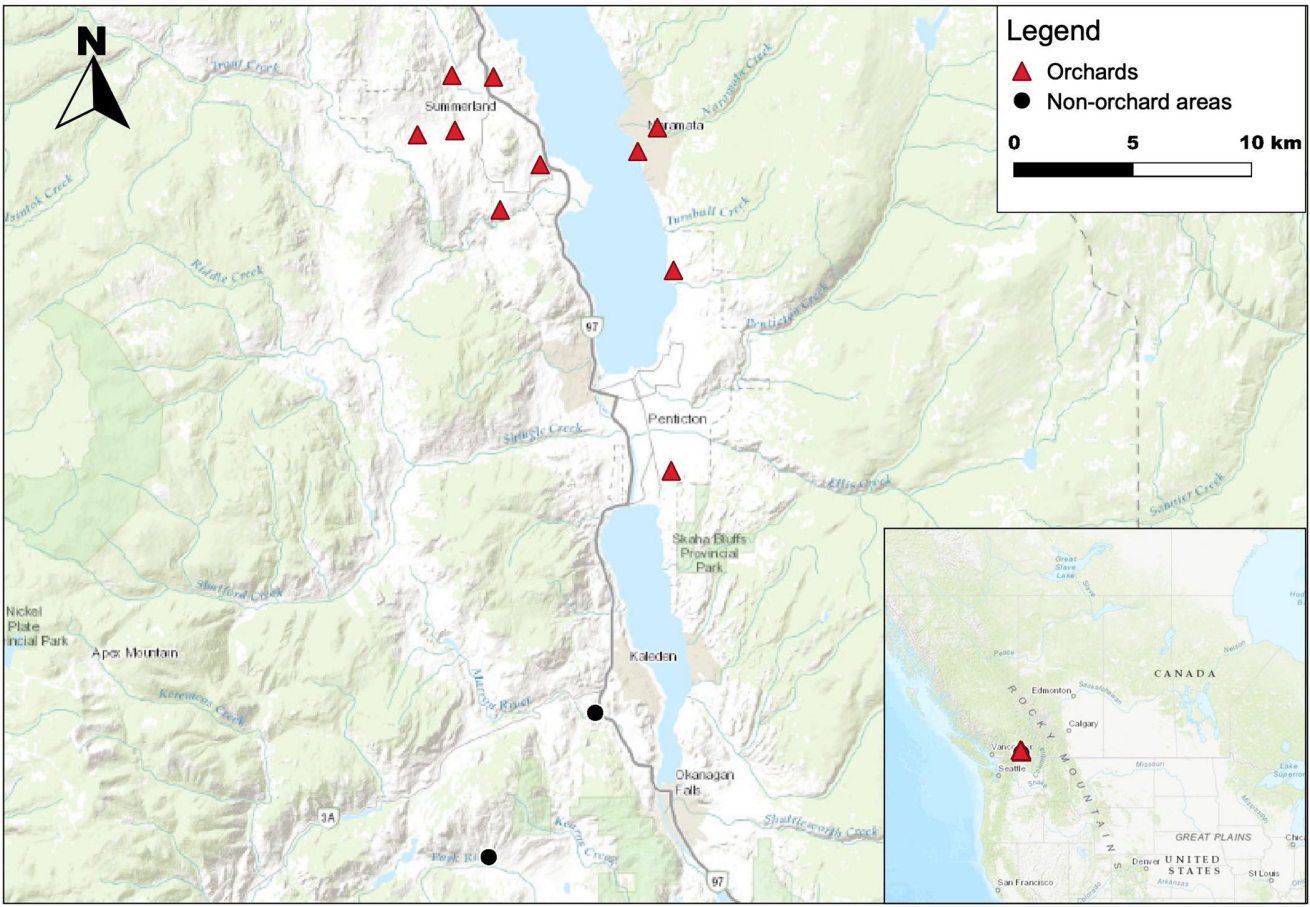
Location of fruit orchards and non‐orchard areas (reference sites) sampled in the Okanagan valley, BC, Canada, 2019.

Sampled fruit orchards were located in the Okanagan valley, British Columbia, Canada, and were within a 20 km^2^ radius of Penticton, Naramata, and Summerland (Figure 1). Ten orchards were sampled from May 13 to June 7, 2019, which included standard‐size, semi‐dwarf and dwarf trees of apple, cherry, and pear varieties. Three out of the 10 orchards were part of the original Harris et al. (2000) study and all other orchards were confirmed to be intact since the 1950s when DDT was actively used. Eggs were also collected from non‐orchard habitats (reference sites), which included dry grassland, open pine forest, and wetlands of the White Lake Grasslands Protected Area, which had no known prior usage of DDT in the area.

### Stable isotope analyses

Stable isotopes of nitrogen (δ^15^N) were used to provide information on trophic position because δ^15^N values in body tissues increase systematically (3–5‰) for each trophic level due to the preferential excretion of lighter amine groups. Stable isotopes of carbon (δ^13^C) were used to characterize diet and habitat use because stable isotopes of carbon during photosynthesis causes C_4_ (higher δ^13^C values) and C_3_ (lower δ^13^C values) plants to have distinct carbon‐isotope (^13^C/^12^C) signatures (Gagnon & Hobson, 2009). Stable isotopes for earthworms and American robin eggs were analyzed at the Ján Veizer Stable Isotope Laboratory at the University of Ottawa. Details about stable isotope analyses are included in the Supporting Information.

### Soil sampling

Three soil samples were collected within each orchard (*n* = 30) with the assumption that there could be spatial differences in contamination (Stringer et al., 1974). Soil samples were collected concurrently and near earthworm sampling locations by digging out a block of soil 0–10 cm deep with a trowel/bulb planter that was cleaned and rinsed with 70% ethanol prior to each use. Each soil sample was transferred to a 250 ml chemically rinsed (acetone/hexane) jar, stored immediately on ice at the site, and within 1–2 h stored frozen at −40 °C until analysis.

### Earthworm sampling

Three earthworm samples were collected in each orchard (*n* = 437 individuals) that were located near active American robin nests with collection sites based on foraging observations. In areas where no foraging was observed near the nests, earthworm samples were collected in damp/moist areas with saturated soil as these locations often represent ideal areas where American robins may forage (Vanderhoff et al., 2020). Earthworms were collected by chemical expulsion with allyl isothiocyanate (AITC; CAS# 57‐06‐7; Fisher Scientific), which is considered to be more effective than other chemical extractants that do not recover all earthworm species equally (Zaborski, 2003). A 4 g/L stock solution was made by diluting AITC with isopropanol (100%; density 0.785; Fisher Scientific). A final amount of 100 mg/L of the stock solution was mixed with 10 L of water and poured over a 60 cm^2^ quadrat with a watering can. Surfacing earthworms were sorted by hand, rinsed in clean water for 60 s, and placed on moist paper towels in aluminum trays for 48 h to clear their gut contents. Earthworms were pooled by species, weighed, and tallied by site before being placed into 100 ml chemically rinsed (acetone/hexane) jars and stored frozen at −40 °C until analysis.

### American robin egg sampling

American robin eggs (*n* = 22 eggs) were located using a systematic row‐by‐row search in nine conventional orchards and one organic orchard. One robin egg was arbitrarily collected from an active nest (i.e. three eggs) for up to three different nests per orchard. Each egg was weighed and measured with a ruler to determine the length, then lightly scored along the circumference with a chemically rinsed (70% ethanol solution) scalpel. Internal egg contents were stored in chemically rinsed (acetone/hexane) jars and frozen at −40 °C until analysis. Previous research has shown that monitoring robin nests and handling eggs does not interfere with overall nest success and has little population impact (Furness, 1993). Handling and collection of American robin eggs was conducted under a Simon Fraser University Animal Care permit (1299B‐19) following guidelines of the Canadian Council on Animal Care and authorized by Environment and Climate Change Canada under a *Migratory Birds Convention Act* permit (SC‐BC‐2019‐0008).

### Chemical analyses

American robin eggs and earthworm samples were shipped frozen on dry ice to the National Wildlife Research Centre in Ottawa, ON and analysed for DDT‐r and other legacy persistent organic pollutants (POPs) using gas chromatography triple quadrupole tandem mass spectrophotometry. Soil samples were shipped frozen on dry ice and analyzed at the Great Lakes Institute for Environmental Research in Windsor, ON. Details about chemical analyses, standards, and quality control/assurance are provided in the Supporting Information.

### Lipid normalization and fugacity

Lipid content in earthworms and robin eggs were determined gravimetrically. To account for differences in lipid content, chemical concentrations in wet weight were expressed in terms of lipid equivalent concentrations (C_*lipid‐equiv*._; µg/g of lipid equivalent). Non‐lipid organic matter was incorporated into the lipid normalization to account for earthworms with low lipid content and high organic carbon content (deBruyn & Gobas, 2009). Dry weight concentrations for soil were expressed as organic carbon‐lipid equivalent fractions (C_*OC‐equiv*._; µg/g of organic carbon lipid equivalent). Chemical activities were also expressed as chemical fugacities to facilitate the comparison of DDT‐r concentrations between abiotic (i.e. soil) and biotic media. Fugacity (*f*; Pa) is defined as the partial pressure that a chemical exerts within a matrix and is determined from the measured concentration of the chemical (*C*; mol/m^3^) and the fugacity capacity (*Z*; mol/Pa·m^3^) of the chemical in the medium as *C*/*Z*. Lipid normalization and fugacity details are included in the Supporting Information.

### Bioaccumulation metrics

Biota‐soil‐accumulation factors (g_OC_/g_lipid_) were calculated by dividing the average lipid normalized concentration of DDT‐r in earthworms by the average organic carbon normalized concentration of DDT‐r in soil. Biota‐soil‐accumulation factor values greater than 1 generally indicates that a chemical is bioaccumulating from soil to biota. Biomagnification factors (robin egg g_lipid_/diet g_lipid_) were calculated by dividing the average lipid normalized concentration of DDT‐r in American robin eggs by the average lipid normalized concentration of DDT‐r in earthworms. Biomagnification factor values greater than 1 indicates that a chemical is biomagnifying, a value less than 1 indicates that a chemical is not biomagnifying (i.e. biodilution), and a value equal to 1 indicates that on average the chemical is likely not biomagnifying (Van den Brink et al., 2015).

### Statistical analyses

All statistical analyses were conducted in R (RStudio, Ver 1.2.5042). Data were tested and confirmed for normality using the Shapiro–Wilk normality test and by inspecting *q*–*q* plots. A one‐way analysis of variance (ANOVA) was used to test for differences in soil DDT‐r concentrations and orchard type. To determine whether different earthworm species contributed to different DDT‐r levels, each DDT‐r compound was analyzed separately as the dependent variable in a mixed effects model with species as a covariate along with density and biomass as main effects and site as a random effect. A one‐way ANOVA was used to determine whether earthworm DDT‐r concentrations were dependent on % soil moisture and % soil organic carbon. Dichlorodiphenyldichlorethane) (1,1,1‐trichloro‐2,2‐bis(p‐chlorophenyl)ethane)‐related levels in robin eggs were analyzed separately as the dependent variable in a mixed effects model with % lipid, developmental stage, and the interaction term egg weight × length as main effects and site as a random effect. Stable isotopes of δ^15^N and δ^13^C were compared between species groups using a mixed effects model with each stable isotope analyzed separately as the dependent variable with species as a covariate and site as a random effect.

Chemical fugacities in different media were analyzed using a one‐way ANOVA and a Tukey's multiple comparisons test (R Core Team, 2019) was conducted if models identified any significant effects. For chemical concentrations below the Method Detection Limit, average concentrations and standard error (SE) for each contaminant were calculated using the Kaplan–Meier model in the Nondetects and Data Analysis of Environmental Data package in the R program (Lee, 2017). Statistical significance of *p*‐values were assessed at *α* = 0.05.

## RESULTS

### Stable isotopes

**Figure 2:**
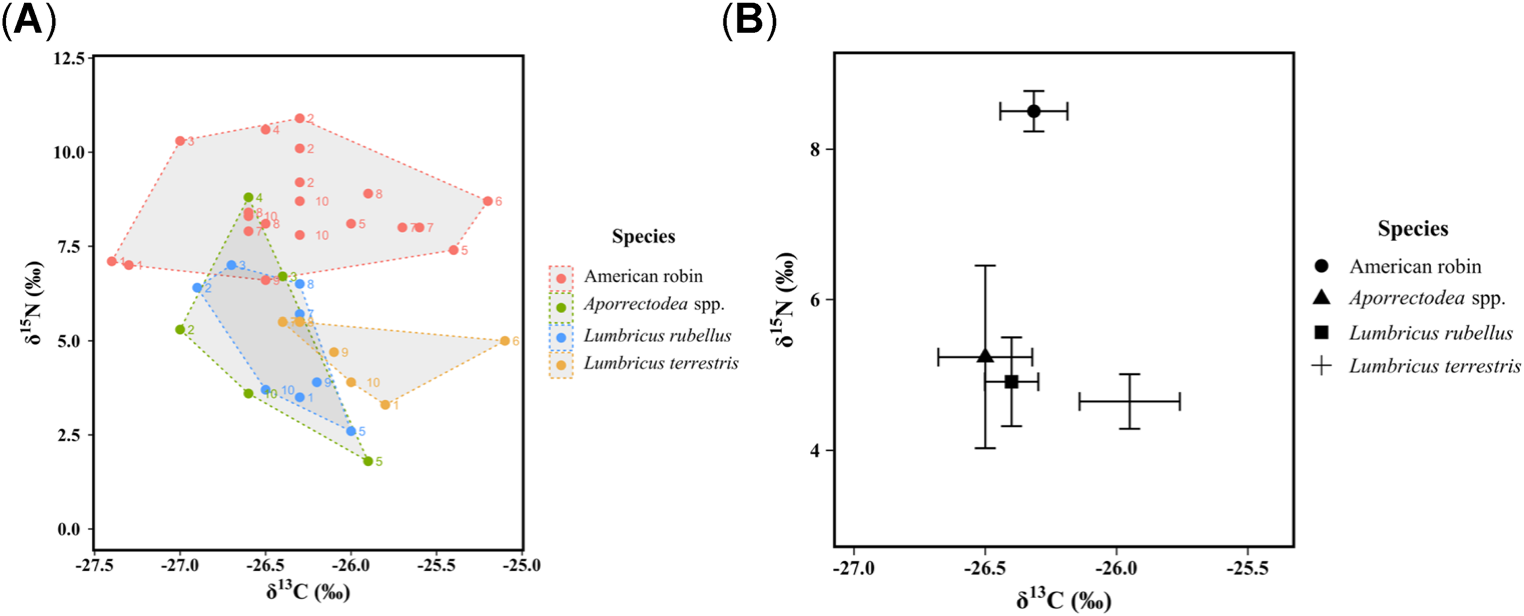
(**A**) Population niche of individual δ^15^N and δ^13^C isotope signatures of American robin (*Turdus migratorius*) eggs (*n* = 22) and earthworm species from Okanagan valley orchards, BC, Canada, 2019. Coloured numbers beside data points represent the different orchards sampled. (**B**) Average stable δ^15^N and δ^13^C isotope biplot of American robin eggs (*n* = 22) and earthworms from Okanagan valley orchards, 2019. Error bars represent the standard error.

Delta^15^N varied between species groups (Figure 2A; *F*_3,27_ = 73.3, *p* < 0.001), with average δ^15^N values ranging from 8.51‰ (±0.25‰; SE) in American robin eggs to 4.65, 4.91, and 5.24‰ in *Lumbricus terrestris*, *Lumbricus rubellus*, and *Aporrectodea* spp., respectively (Figure 2B). The average δ^15^N value for all earthworms was 4.92‰ (±0.72‰), which was not statistically different among earthworm species (*F*_2,7_ = 1.88, *p* > 0.1). Delta^13^C did not vary between species groups (*F*_3,28_ = 1.41, *p* > 0.1) and there was considerable overlap in the δ^13^C values in our food chain, with average δ^13^C values ranging from −26.32‰ (±0.12‰) in American robin eggs to −25.95, −26.40, and −26.50‰ in *L. terrestris*, *L. rubellus*, and *Aporrectodea* spp., respectively. The average δ^13^C value for all earthworms was −26.28‰ (±0.16‰), with average δ^13^C values being statistically higher in *L. terrestris* than *L. rubellus* (*F*_2,7_ = 3.40, *p* < 0.05).

### Soil

Concentrations of DDT‐r in soil samples (*n* = 30) collected from Okanagan valley fruit orchards ranged over several orders of magnitude (Table 1). Across orchards, *p,p*′‐DDE and *p,p*′‐DDT were the most dominant compounds in soil, comprising an average of 54 and 44% of ∑DDT, respectively, versus 2.1% for dichlorodiphenyldichloroethane (*p,p*′‐DDD). The maximum detected concentrations of *p,p*′‐DDE and *p,p*′‐DDT were 89.2 and 116 µg/g (organic carbon lipid equiv.), respectively, in apple orchards, while those of *p,p*′‐DDD were less variable and peaked 0.749 µg/g in a cherry orchard. Soil DDE:DDT ratios were highly variable across the region and some orchards had values <1 suggesting a more recent use of DDT. We did not observe a statistical difference in contamination with orchard type (i.e. apple, cherry, pear) for either *p,p*′‐DDE (*F*_2,14_ = 0.624, *p* > 0.1), *p,p*′‐DDT (*F*_2,23_ = 0.621, *p* > 0.1), or *p,p*′‐DDD (*F*_2,8_ = 0.494, *p* > 0.1) concentrations in soil.

**Table 1:** Dichlorodiphenyldichlorethane‐related concentrations in soil samples (0–10 cm) from Okanagan valley fruit orchards, BC, Canada, 2019^a^

				Mean concentration (µg/g organic carbon‐lipid equivalent)			
Orchard	Mass^b^	% moisture	% organic carbon	*p,p′‐*DDE	*p,p′‐*DDT	*p,p′*‐DDD	DDE:DDT
Cherry orchard—1	16.5	17.5	21.2	0.274	0.100	0.062	2.75
		(7.60–25.9)	(9.08–31.0)	(nd^c^–0.690)	(nd–0.244)	(nd–0.123)	
Apple orchard—2	15.0	25.1	36.5	1.89	3.55	0.101	0.532
		(19.3–29.8)	(36.1–36.7)	(nd^c^–2.25)	(nd–4.59)	(nd–0.159)	
Pear orchard—3	16.8	16.0	26.1	19.7	11.7	0.182	1.68
		(8.74–25.6)	(12.5–33.4)	(5.62–31.0)	(3.15–28.3)	(0.172–0.197)	
Apple orchard—4	16.1	19.7	29.0	21.3	10.0	0.130	2.13
		(6.17–43.8)	(11.5–60.2)	(6.95–47.0)	(3.90–20.2)	(0.038–0.258)	
Cherry orchard—5	16.6	17.0	21.0	11.6	7.84	0.089	1.48
		(14.8–19.6)	(18.8–23.5)	(4.11–18.2)	(0.738–11.9)	(0.046–0.146)	
Apple orchard—6	15.5	22.7	31.4	2.46	1.23	0.023	2.00
		(18.8–25.3)	(26.6–40.1)	(1.05–4.76)	(0.348–2.16)	(0.014–0.028)	
Apple orchard—7	19.3	3.70	11.4	63.7	87.1	0.524	0.731
		(2.99–4.85)	(6.67–18.2)	(40.1–89.2)	(59.2–116)	(0.409–0.615)	
Apple orchard—8	17.0	15.3	22.4	14.0	11.5	0.135	1.22
		(2.62–22.4)	(11.8–28.4)	(1.39–30.0)	(0.382–23.1)	(0.021–0.225)	
Cherry orchard—9	15.6	22.1	33.0	13.4	13.4	0.454	1.00
		(13.8–30.1)	(24.0–43.0)	(7.19–19.0)	(4.40–20.5)	(0.130–0.749)	
Cherry orchard—10	17.0	15.0	18.0	12.5	18.7	0.160	0.668
		(6.51–20.8)	(7.79–24.7)	(5.34–16.6)	(2.83–33.3)	(0.086–0.293)	
Orchard mean^d^	16.5 ± 0.35	17.4 ± 1.8	25.0 ± 2.2	16.1 ± 5.8	16.5 ± 8.1	0.186 ± 0.053	1.42 ± 0.23

^a^ Values in parentheses are ranges.

^b^ Mass of each soil sample; measured in grams.

^c^ One soil subsample contained DDT‐r concentrations below the Method Detection Limit (MDL). The mean DDT‐r concentration in these orchards were calculated using a Kaplan–Meier (KM) statistical model.

^d^ Average value across all orchards; expressed as mean ± standard error.

Dichlorodiphenyldichloroethylene (1,1‐dichloro‐2,2‐bis(4‐chlorophenyl)ethylene = DDE; dichlorodiphenyldichlorethane (1,1,1‐trichloro‐2,2‐bis(p‐chlorophenyl)ethane = DDT; DDT‐r = DDT‐related; dichlorodiphenyldichloroethane = DDD.

### Earthworms

There was a 120‐fold difference in *p,p′‐*DDE concentrations across earthworms, with concentrations ranging from 2.41 to 288 µg/g in a *L. terrestris* sample from an apple orchard (Table 2). Based on a mixed effects model, mean *p,p′‐*DDE concentrations were statistically higher in *L. terrestris* at 117 µg/g (39 SE) compared to *L. rubellus* at 77 µg/g (17 SE; *p* < 0.05); however, there were no statistical differences in mean *p,p′‐*DDE concentrations between *Aporrectodea* spp. (mean 103 µg/g; 12 SE) and *Lumbricidae* spp. (*p* > 0.05). Mean *p,p′‐*DDT concentrations were not statistically different between *Aporrectodea* spp. at 23 µg/g (4.9 SE) and *Lumbricidae* spp. at 17 µg/g (4.7 SE; *p* > 0.1). Mean *p,p′‐*DDD concentrations were statistically higher in *L. terrestris* at 5.1 µg/g (2.7 SE) compared to *Aporrectodea* spp. at 2.4 µg/g (0.72 SE; *p* < 0.05) and *L. rubellus* at 2.9 µg/g (1.1 SE; *p* < 0.05).

**Table 2:** Dichlorodiphenyldichlorethane (1,1,1‐trichloro‐2,2‐bis(p‐chlorophenyl)ethane‐related concentrations in *Lumbricidae* and *Aporrectodea* earthworm samples collected from Okanagan valley fruit orchards, BC, Canada, 2019

						Mean concentration (µg/g lipid‐equivalent)			
Species	Location	Total^a^	Mass^b^	% moisture	% lipid	*p,p′‐*DDE	*p,p′‐*DDD	*p,p′*‐DDT	DDE:DDT
*Lumbricus terrestris*	Cherry orchard—1	4	25.4	90.4	0.59	2.41	0.096	0.711	3.39
*Lumbricus rubellus*	Cherry orchard—1	28	18.7	86.3	1.19	12.7	0.314	4.45	2.84
*Lumbricus rubellus*	Apple orchard—2	60	38.8	84.9	0.77	170	4.79	42.9	3.97
*Aporrectodea* spp.	Apple orchard—2	4	10.6	86.3	0.26	76.3	2.15	24.8	3.08
*Lumbricus rubellus*	Pear orchard—3	73	56.2	86.2	1.12	102	3.56	28.6	3.57
*Aporrectodea* spp.	Pear orchard—3	13	9.16	86.5	0.59	147	5.08	41.2	3.58
*Aporrectodea* spp.	Apple orchard—4	8	6.14	85.1	0.39	86.7	0.923	5.41	16.0
*Lumbricus rubellus*	Cherry orchard—5	25	17.8	76.3	1.23^c^	65.3	1.05	15.4	4.23
*Aporrectodea* spp.	Cherry orchard—5	15	5.32	81.8	0.79	100	1.49	20.9	4.78
*Lumbricus terrestris*	Apple orchard—6	6	19.8	86.0	0.32	88.1	2.14	22.1	3.99
*Lumbricus rubellus*	Apple orchard—6	47	30.4	86.6	0.94	36.2	1.51	5.72	6.33
*Lumbricus terrestris*	Apple orchard—7	6	25.9	86.0	1.11	288	18.5	25.3	11.4
*Lumbricus terrestris*	Apple orchard—8	9	50.1	87.0	1.38	83.1	2.78	7.80	10.7
*Lumbricus rubellus*	Apple orchard—8	41	22.4	80.7	1.64	95.5	9.47	5.71	16.7
*Lumbricus terrestris*	Cherry orchard—9	14	33.8	94.7	1.23	106	3.54	19.7	5.37
*Lumbricus rubellus*	Cherry orchard—9	40	18.9	85.1	1.42	53.0	1.35	11.1	4.76
*Lumbricus terrestris*	Cherry orchard—10	3	11.2	91.9	1.22	133	3.79	31.7	4.19
*Lumbricus rubellus*	Cherry orchard—10	18	9.61	85.4	1.56	79.1	1.58	18.3	4.32
*Aporrectodea* spp.	Cherry orchard—10	23	7.97	84.7	1.32	108	2.29	24.1	4.49
Species mean		23 ± 4.7	22.0 ± 3.3	85.8 ± 0.89	0.99 ± 0.10	96.5 ± 14.3	3.50 ± 0.964	18.7 ± 2.88	6.20 ± 0.98

^a^ Total count is defined as the total number of individual earthworms collected within a 60 cm^2^ quadrat across sampling sites within the orchard.

^b^ Total weight of earthworm species collected across sampling sites within the orchard; measured in grams.

^c^ % lipid could not be calculated for this sample due to an unexpected lab issue and was approximated using an arithmetic mean value for that species.

Dichlorodiphenyldichloroethylene (1,1‐dichloro‐2,2‐bis(4‐chlorophenyl)ethylene = DDE; dichlorodiphenyldichlorethane (1,1,1‐trichloro‐2,2‐bis(p‐chlorophenyl)ethane = DDT; dichlorodiphenyldichloroethane = DDD.

There was evidence of a negative effect of earthworm density on the mean concentration of *p,p′‐*DDD (*F*_1,5_ = 7.87, *p* < 0.05), but not on the mean concentration of *p,p′‐*DDE (*F*_1,5_ = 3.57, *p* > 0.1) and *p,p*′‐DDT (*F*_1,5_ = 2.79, *p* > 0.1). Soil moisture content (%) appeared to be inversely related to mean *p,p*′‐DDE (*F*_1,8_ = 8.34, *p* < 0.05) and *p,p*′‐DDD (*F*_1,8_ = 15.2, *p* < 0.01) concentrations in earthworms. Soil organic carbon (%) had no statistical effect on mean *p,p*′‐DDE (*F*_1,8_ = 2.21, *p* > 0.1), *p,p*′‐DDT (*F*_1,8_ = 0.03, *p* > 0.1), or *p,p′*‐DDD (*F*_1,8_ = 3.99, *p* < 0.1) concentrations in earthworms. ∑DDT concentrations in earthworms were not correlated with δ^15^N (*F*_1,11_ = 0.88, *p* > 0.1) or δ^13^C (*F*_1,17_ = 3.55, *p* < 0.1) values in earthworm tissue.

### American robin eggs

The average weight and length of American robin eggs in our study were 6.44 ± 0.16 g and 2.74 ± 0.04 cm in orchards, and 7.15 ± 0.65 g and 2.85 ± 0.1 cm in non‐orchard areas, respectively. On a wet weight basis, *p,p*′‐DDE concentrations in robin eggs were independent of the weight × length interaction term (*F*_1,15_ = 0.28, *p* > 0.1) and were not related to % egg moisture (*F*_1,11_ = 0.0009, *p* > 0.1). Dichlorodiphenyldichlorethane (1,1,1‐trichloro‐2,2‐bis(p‐chlorophenyl)ethane)‐related concentrations were highly variable among individual robin eggs (Table 3), which averaged 36.6, 1.14, and 0.156 µg/g (wet wt) or 568, 19, and 2.5 µg/g (lipid equiv.) for *p,p*′‐DDE, *p,p*′‐DDT, and *p,p*′‐DDD, respectively. The highest *p,p*′‐DDE concentration detected was 107 µg/g (wet wt) or 1,979 µg/g (lipid equiv.) in an egg from an apple orchard. Concentrations of *p,p*′‐DDE in American robin eggs were statistically higher in orchards versus non‐orchard areas (*F*_1,20_ = 3.95, *p* < 0.05). Concentrations of ∑DDT in robin eggs were not correlated with δ^15^N (*F*_1,16_ = 0.44, *p* > 0.1) or δ^13^C (*F*_1,19_ = 0.19, *p* > 0.1) values in eggs. In orchards 6, 7, 8, and 10, female shifts in δ^13^C were consistent with an increase in *L. terrestris* in the diet during egg laying. Aside from DDT‐r, several other halogenated contaminants were detected in our American robin eggs, although they were not particularly elevated (Table 4).

**Table 3:** Dichlorodiphenyldichlorethane (1,1,1‐trichloro‐2,2‐bis(p‐chlorophenyl)ethane‐related concentrations in American robin (*Turdus migratorius*) eggs collected from fruit orchards and non‐orchard areas (White Lake) in the Okanagan valley, BC, Canada, 2019^a^

			Mean concentration (µg/g lipid‐equivalent)			
Location	% moisture	% lipid	*p,p*′‐DDE	*p,p*′‐DDT	*p,p*′‐DDD	DDE:DDT
Cherry orchard—1	80.4	5.76	208	0.467	0.052	445
	(80.2–80.6)	(5.50–6.02)	(207–209)	(0.331–0.602)	(0.040–0.064)	
Apple orchard—2	82.8	4.62	849	27.7	3.94	30.6
	(82.4–83.6)	(4.17–5.18)	(457–1464)	(18.1–43.1)	(1.86–6.48)	
Pear orchard—3	82.6	5.80	578	27.7	3.31	20.8
Apple orchard—4	81.8	5.84	429	19.2	1.35	22.3
Cherry orchard—5	83.4	5.45	213	6.93	0.546	30.7
	(82.9–83.8)	(5.31–5.58)	(182–244)	(4.73–9.14)	(0.440–0.651)	
Apple orchard—6	82.4	4.91	59.2	3.32	0.390	17.8
Apple orchard—7	82.5	4.86	1110	29.2	5.37	38.0
	(81.6–83.7)	(4.29–5.54)	(631–1979)	(23.5–37.5)	(2.65–8.51)	
Apple orchard—8	83.4	4.21	709	22.2	2.99	32.0
	(83.0–83.9)	(3.55–4.74)	(543–796)	(19.8–26.1)	(1.75–3.77)	
Cherry orchard—9	81.9	5.63	652	24.3	2.96	26.8
Cherry orchard—10	84.0	5.32	870	28.3	3.70	30.7
	(83.3–84.6)	(5.04–5.86)	(464–1239)	(19.3–35.4)	(2.02–5.88)	
Orchard mean^b^	82.5 ± 0.32	5.24 ± 0.18	568 ± 107	18.9 ± 3.52	2.46 ± 0.562	69.5 ± 41.8
Non‐orchard	82.6	5.80	7.87	0.407	0.025	16.75
	(82.4–82.7)	(5.65–5.95)	(2.16–13.6)	(0.180–0.635)	(0.009–0.042)	

^a^ Values in parentheses are ranges.

^b^ Average value across all sites; expressed as mean ± standard error.

Dichlorodiphenyldichloroethylene (1,1‐dichloro‐2,2‐bis(4‐chlorophenyl)ethylene = DDE; dichlorodiphenyldichlorethane (1,1,1‐trichloro‐2,2‐bis(p‐chlorophenyl)ethane = DDT; dichlorodiphenyldichloroethane = DDD.

**Table 4:** Presence of other organochlorines, polychlorinated biphenyls (PCBs) and polybrominated diphenyl ethers (PBDEs) in American robin (*Turdus migratorius*) eggs collected from Okanagan valley fruit orchards, BC, Canada, 2019^a^

Compound	Concentration	%^b^
Oxychlordane	0.002	55
Trans‐nonachlor	0.004	65
Hexachlorobenzene	0.001	45
Heptachlor epoxide	0.001	25
Dieldrin	0.011	80
PCB‐138	0.002	100
PCB‐153	0.002	80
PCB‐180	0.001	75
PCB‐187	0.002	100
PBDE‐47	3.68 ± 0.57	100
PBDE‐49	0.113 ± 0.01	20
PBDE‐85	0.147 ± 0.02	20
PBDE‐99	5.48 ± 1.1	100
PBDE‐100	2.42 ± 0.52	100
PBDE‐153	0.866 ± 0.15	100
PBDE‐154/153	0.454 ± 0.09	95
∑HCH^c^	nd^f^	–
∑Mirex^d^	nd	–
∑OCS^e^	nd	–

^a^ Mean concentrations for organochlorines and PCBs are measured in µg/g (wet wt) and those for PBDEs are measured in ng/g (wet wt); concentrations for PBDEs are expressed as means ± standard error.

^b^ %, % of robin eggs with detectable levels based on the Method Detection Limit reported for each compound.

^c^ ∑Hexachlorocyclohexane (∑HCH): α‐hexachlorocyclohexane, β‐hexachlorocyclohexane, and γ‐hexachlorocyclohexane.

^d^ ∑Mirex: mirex and photomirex.

^e^ ∑Octachlorostyrene (∑OCS).

^f^ Not detected based on the Method of Detection Limit.

### Bioaccumulation of DDT‐r in American robin orchard food chains

**Figure 3:**
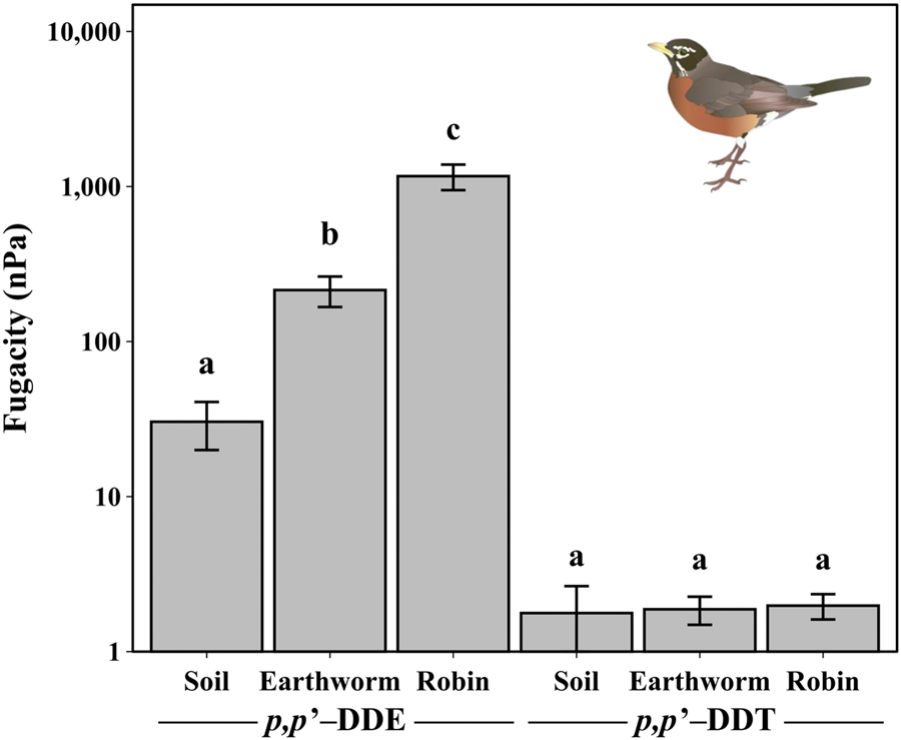
Comparison of the mean fugacity (nPa) of dichlorodiphenyldichloroethylene (1,1‐dichloro‐2,2‐bis(4‐chlorophenyl)ethylene) (*p,p*′‐DDE) and dichlorodiphenyldichlorethane (1,1,1‐trichloro‐2,2‐bis(p‐chlorophenyl)ethane) (*p,p*′‐DDT) in the American robin (*Turdus migratorius*) orchard food chain. Error bars represent the standard error. Letters above the bars denote significance among the sampling media for that compound.

Mean fugacities for *p,p*′‐DDE in soil, earthworms, and American robin eggs were 30.4 nanopascal (nPa; 10.4 SE), 215 nPa (48 SE), and 1166 nPa (218 SE), respectively, and were statistically different from each other (*F*_2,27_ = 22.3, *p* < 0.001), suggesting that an increase in thermodynamic potential occurs as *p,p*′‐DDE is transferred from prey to predator (Figure 3). By contrast, mean fugacities for *p,p*′‐DDT in soil, earthworms, and American robin eggs were 1.77 nPa (0.87 SE), 1.87 nPa (0.39 SE), and 1.98 nPa (0.37 SE), respectively, and were not statistically different from each other (*F*_2,27_ = 0.03, *p* > 0.1), suggesting that the fugacities for *p,p*′‐DDT in these environmental media were relatively close or in equilibrium and that *p*,*p*'‐DDT is likely not biomagnifying in our food chain (Figure 3).

Biota‐soil‐accumulation factors for DDT‐r ranged from 0.29 to 79.1 and across 10 orchards, averaged 16.1, 5.59, and 26.4 g_OC_/g_lipid_ for *p,p*′‐DDE, *p,p*′‐DDT, and *p,p*′‐DDD, respectively (Table 5), indicating that aged DDT‐r was still bioaccumulating in earthworms via dietary exposure. In orchards 4, 7, and 8, BSAFs for *p,p*′‐DDT were <1, indicating that chemical bioavailability of *p,p*′‐DDT was limited in these orchards. Biomagnification factors ranged over several orders of magnitude and across 10 orchards, averaged 7.57, 1.31, and 0.77 g_lipid_/g_lipid_ for *p,p*′‐DDE, *p,p*′‐DDT, and *p,p*′‐DDD, respectively (Table 6). In orchard 6, the BMF for *p,p*′‐DDE was <1, suggesting that the female robin was foraging either in a non‐contaminated area or non‐orchard habitat(s), and/or feeding on non‐contaminated prey. Collectively, these BSAF and BMF indices suggest that *p,p*′‐DDE has a greater biomagnification capacity in our American robin food chain than *p,p*′‐DDT and *p,p*′‐DDD.

**Table 5:** Biota‐soil‐accumulation factors (BSAFs) from soil to earthworms in Okanagan valley orchards, BC, Canada, 2019^a^

		BSAF (g_OC_/g_lipid_)		
Location	Type	*p,p*′‐DDE	*p,p*′‐DDT	*p,p*′‐DDD
Orchard 1	Cherry	27.5	25.9	3.29
Orchard 2	Apple	65.2	9.52	34.5
Orchard 3	Pear	6.32	2.97	23.7
Orchard 4	Apple	4.08	0.539	7.10
Orchard 5	Cherry	7.10	2.32	14.31
Orchard 6	Apple	25.2	11.3	79.1
Orchard 7	Apple	4.53	0.290	35.4
Orchard 8	Apple	6.38	0.588	45.4
Orchard 9	Cherry	5.92	1.16	5.39
Orchard 10	Cherry	8.55	1.32	16.0
Average^b^		16.1 ± 6.1	5.59 ± 2.6	26.4 ± 7.4

^a^ BSAFs were calculated using organic carbon‐lipid equivalent concentrations (µg/g OC‐equiv.) for soil and lipid equivalent concentrations (µg/g lipid equiv.) for earthworms from each orchard.

^b^ Average BSAF across all orchards; expressed as mean ± standard error.

Dichlorodiphenyldichloroethylene (1,1‐dichloro‐2,2‐bis(4‐chlorophenyl)ethylene = DDE; dichlorodiphenyldichlorethane (1,1,1‐trichloro‐2,2‐bis(p‐chlorophenyl)ethane = DDT; dichlorodiphenyldichloroethane = DDD.

**Table 6:** Biomagnification factors (BMFs) from earthworms to American robin (*Turdus migratorius*) eggs in Okanagan valley orchards, BC, Canada, 2019^a^

		BMF (robin g_lipid_/diet g_lipid_)		
Location	Type	*p,p*'‐DDE	*p,p*'‐DDT	*p,p*'‐DDD
Orchard 1	Cherry	28.0	0.181	0.255
Orchard 2	Apple	6.89	0.820	1.14
Orchard 3	Pear	4.64	0.795	0.766
Orchard 4	Apple	4.95	3.55	1.47
Orchard 5	Cherry	2.58	0.382	0.430
Orchard 6	Apple	0.951	0.238	0.213
Orchard 7	Apple	3.85	1.15	0.290
Orchard 8	Apple	7.94	3.28	0.488
Orchard 9	Cherry	8.20	1.58	1.21
Orchard 10	Cherry	8.16	1.15	1.45
Average^b^		7.57 ± 2.4	1.31 ± 0.38	0.77 ± 0.16

^a^ BMFs were calculated using lipid equivalent concentrations (µg/g lipid equiv.) for earthworms and lipid equivalent concentrations (µg/g lipid equiv.) for American robin eggs from each orchard.

^b^ Average BMF across all orchards; expressed as mean ± standard error.

Dichlorodiphenyldichloroethylene (1,1‐dichloro‐2,2‐bis(4‐chlorophenyl)ethylene = DDE; dichlorodiphenyldichlorethane (1,1,1‐trichloro‐2,2‐bis(p‐chlorophenyl)ethane = DDT; dichlorodiphenyldichloroethane = DDD.

## DISCUSSION

### DDT‐r and other halogenated chemicals in American robin eggs

In the present study, we found detectable levels of DDT‐r in every American robin egg collected from Okanagan valley orchards with average concentrations of *p,p*′‐DDE, *p,p*′‐DDT, and *p,p*′‐DDD in eggs at 36.6, 1.14, and 0.156 µg/g (wet wt) or 568, 18.9, and 2.46 µg/g (lipid eq.), respectively. Our BSAFs and BMFs calculated for *p,p*′‐DDE were greater than 1 in nearly every orchard sampled and our fugacity analyses suggested that *p,p*′‐DDE was still thermodynamically biomagnifying in American robin food chains 26 years after a previous study. On a wet weight basis, mean *p,p*′‐DDE concentrations in our eggs showed a 2.3‐fold decrease relative to the mean *p,p*′‐DDE concentrations in robin eggs reported by Harris et al. (2000) in the same region, which averaged 85 µg/g. Our robin eggs from Penticton, Naramata, and Summerland fruit orchards also had lower ∑DDT concentrations (mean 38 µg/g) compared to robin eggs collected from orchards between 1993 to 1998 in the same regional districts, which averaged 49 µg/g (Gill et al., 2003) and 68 µg/g (Iwaniuk et al., 2006), indicating that although contamination is still present, DDT‐r levels have slightly decreased over the years.

Dichlorodiphenyldichlorethane (1,1,1‐trichloro‐2,2‐bis(p‐chlorophenyl)ethane)‐related concentrations in Okanagan valley robin eggs were higher when compared to reported values in robin eggs and other passerines from other North American agroecosystems. For instance, mean *p,p*′‐DDE concentrations in our robin eggs were three times higher than in robin eggs sampled from orchards in Washington State where up to 73 kg DDT/ha was applied annually (Blus et al., 1987), and 1.5 times higher than in robin eggs from orchards in southwestern Ontario (Hebert et al., 1994), which was sampled over three decades ago. Interestingly, mean *p,p*′‐DDE concentrations in our robin eggs were up to three times higher than in robin eggs and nestlings from apple, cherry, and peach orchards in New York's Hudson River Valley in the 1960s where approximately 45 kg DDT/ha/year was applied continually for twenty years (Johnson et al., 1976). In further comparisons, mean *p,p*′‐DDE concentrations in our robin eggs were up to eight times higher than in red‐winged blackbird (*Agelaius phoeniceus*) and tree swallow (*T. bicolor*) eggs from the Great Lakes and St. Lawrence River basin (Bishop et al., 1995), and several orders of magnitude greater than in European starling (*Sturnus vulgaris*) eggs from a rural agricultural site in British Columbia (Eng et al., 2014). These results clearly demonstrate that the higher DDT‐r burdens among Okanagan valley orchard robins are likely due to their principal earthworm diet and ground‐foraging behaviour, compared to other omnivorous and insectivorous birds. Several other halogenated contaminants were detected in our robin eggs, which were well below levels previously reported in robin eggs in the 1990s from the same region (Harris et al., 2000). Thus, clearly among the legacy and more recently released POPs, the DDT‐r compounds stand out starkly as persistent organic contaminants in our agroecosystem.

### DDT‐r contamination in Okanagan valley orchard soils and earthworms

In a survey of untilled fruit orchards, concentrations of ∑DDT fell below detection limits past a 50 cm depth and nearly 100% of ∑DDT concentrations were detected within the top 10 cm of soil (Harris et al., 2000; Stringer et al., 1974) suggesting that our soil samples were a fair representation of DDT‐r contamination. Accordingly, historical DDT application rates and diverse climatic conditions are believed to be the most prevalent factors influencing DDT‐r levels observed in Okanagan valley orchards. For example, during the 1960s, the British Columbia Ministry of Agriculture and Food (1969) recommended that up to 7 kg of *p,p*′‐DDT per hectare be applied two to four times per year, indicating that 28 kg of *p,p*′‐DDT ha year^−1^ could have been sprayed in the Okanagan valley. However, different pest outbreaks in orchards throughout the growing season(s) could have resulted in higher spray concentrations and frequencies and some British Columbia areas may have received more than 90 000 kg of technical DDT over the total period of use (Elliott et al., 1989). Conversely, the Ontario Department of Agriculture (1968) recommended that 3.4 kg/g of *p,p*′‐DDT/ha be applied two to three times per year, hence 10 kg of *p,p*′‐DDT ha year^−1^ could have been sprayed in Ontario orchards during the same time period, thus, amounting to lower surface contamination levels observed in some eastern provinces.

Dichlorodiphenyldichloroethylene (1,1‐dichloro‐2,2‐bis(4‐chlorophenyl)ethylene):DDT ratios have been used as a proxy for the age and rate of transformation of DDT‐r in the environment and our low average ratio of 1.42 is similar to Harris's study of 1.10 at the same surface soil depth (0–10 cm). This indicates that: (1) Okanagan valley orchards received DDT applications later in time, on average; (2) *p,p*′‐DDT has degraded slowly into *p,p*′‐DDE under aerobic conditions (i.e. sandy loam soils), thus prolonging its degradation time and half‐life to ~20–30 years (Harris et al., 2000); (3) *p,p*′‐DDE has been subjected to volatilization as a result of a warmer climate in the interior; and/or (4) a lack of tillage or plowing in orchards reduced the loss of *p,p*′‐DDT (Elliott et al., 1994). Ongoing illicit DDT applications in the Okanagan valley are unlikely given the absence of higher *p,p*′‐DDT levels in our soil and worm samples that otherwise would have been directly associated with sprayed areas and due to many species of insects reportedly developing resistance to DDT (Harris et al., 2000). However, trace metal contamination with synthetic lead arsenates has been reported in some horticultural ecosystems, including fruit orchards where arsenic‐based insecticides were used prior to the introduction of DDT to control Lepidopteran pests (Elfving et al., 1994). A study conducted in New Zealand fruit orchards found statistically positive relationships between aged *p,p*′‐DDE and lead (11–178 µg/g) and arsenic (2–34 µg/g) concentrations in soil and earthworm tissue (Gaw et al., 2012) and there is some evidence showing that these heavy metals can inhibit the microbial degradation of DDT‐r in soil (Gaw et al., 2003). We are currently examining heavy metal levels in Okanagan valley orchards sampled in the present study.

Concentrations of DDT‐r in our earthworms were lower than earthworms collected from agricultural fields in southern and midwestern United States (mean ∑DDT 12.3 µg/g dry wt; Gish, 1970) and apple orchards in coastal states (mean ∑DDT 147.6 µg/g; Kuhr et al., 1974). In Okanagan valley orchards where DDT had been discontinued for approximately 20 years, earthworms contained 43.5 µg/g of *p,p*′‐DDE, 17.2 µg/g of *p,p*′‐DDT, and 2.2 µg/g of *p,p*′‐DDD (Harris et al., 2000). The low *p,p*′‐DDT concentrations coupled with the high DDE:DDT ratios in our earthworms are consistent with previous studies where earthworms biotransformed *p,p*′‐DDT into *p,p*′‐DDE either directly within the gut (Edwards & Jeffs, 1974) and/or indirectly from microbial degradation stimulated by an increase in soil pH, soil moisture, and nutrient content as a result of burrowing and bioturbation activities (Xu et al., 2020). The Okanagan valley is also a semi‐arid region characterized by low annual precipitation, which could inhibit the breakdown of organic matter and prevent the desorption of DDT‐r from the soil matrix (Harris et al., 2000). Yet, if such aging/weathering is significant, then a substantial fraction of soil‐bound DDT‐r would have been inaccessible to earthworms over the years. Our results suggest that *Lumbricidae* and *Aporrectodea* earthworms are still bioaccumulating and retaining aged DDT‐r due to dermal absorption and the ingestion of soil particulates consequently increasing the bioavailability of DDT‐r within their tissues (Verma & Pillai, 1991).

### Bioaccumulation of DDT‐r in American robin orchard food chains

Stable isotope analyses of δ^15^N and δ^13^C have emerged as powerful biogeochemical tracers to investigate trophic relationships and dietary sources within food webs (Elliott et al., 2021; Morrissey et al., 2010; Van den Brink et al., 2015), although few studies have quantified the influence of diet on DDT‐r exposure in American robins. Our egg homogenate δ^15^N values and isotopically heterogenous δ^13^C isoscape indicated that American robins were feeding at similar trophic levels, but on different carbon sources and detrital content. A plausible explanation for this is that robins are simply shifting between different types of earthworms (epigeic, endogeic, anecic) or species of worms in orchards with different historic spatial patterns of contamination and soil component chemical bioavailability rather than feeding on prey from different trophic levels. Gagnon and Hobson (2009) found that American robins retained high blood and claw δ^15^N values year‐round and argued that robins breeding in agricultural environments tend to have δ^15^N‐enriched tissue lipids that is likely due to a detritivore dominated food web high in baseline δ^15^N and/or the use of fertilizers and other agrochemicals that increase soil δ^15^N pools through ammonification. That contrasts with a study by Fremlin et al. (2020) who reported a mean δ^15^N value of 5.30‰ in American robin tissues and 2.59‰ in earthworms from the lower mainland of British Columbia, which was lower than our value of 8.51 and 4.92‰, respectively. Thus, it appears that urban robins are likely feeding on more plant material and/or anthropogenic food items (i.e. fruits/seeds) than robins in Okanagan valley orchard systems or feeding on smaller or juvenile earthworms, which tend to have lower δ^15^N levels than adult worms (Briones et al., 2001). We argue that the biomagnification of *p,p*′‐DDE in American robin food chains is therefore influenced/confounded by various site‐specific environmental conditions.

Earthworm communities in our orchards were dominated by the *Lumbricidae* and *Aporrectodea* families and consisted of different species and ecotypes. In the Okanagan valley, Harris et al. (2000) reported equitable DDT‐r exposure among *Lumbricidae*, *Aporrectodea*, *Eisenia*, and *Octolasion* spp., and Elliott et al. (1994) argued that the feeding/burrowing behaviour of large anecic species, such as *L. terrestris*, could dilute surface soil *p,p*′‐DDE concentrations to other worms. Kelsey et al. (2005) claimed that the uptake of *p,p*′‐DDE was 10‐fold higher in epigeic earthworms compared to endogeic and anecic species, although their study was not specifically designed to assess exposure under natural conditions considering soil was mixed with *p,p*′‐DDE only (and not *p,p*′‐DDT) and because earthworms were tested individually and not simultaneously. In the present study, *Lumbricidae* and *Aporrectodea* species shared a roughly similar δ^15^N value (i.e. 4.65–5.24‰), indicating that there were no interspecific differences in trophic niches. However, our δ^13^C isotope signatures revealed some feeding ecology differences with *L. terrestris* showing a markedly enriched δ^13^C isotope, suggesting preferential assimilation of soil organic matter derived from C_4_ vegetation in a hot and arid agroecosystem (Briones et al., 2001) over C_3_, which is typically consumed by epigeic/endogeic species and is part of a temperate grassland ecosystem (Currier et al., 2020). Associations with soil matrices enriched in δ^13^C may explain why *L. terrestris* and other worms at low trophic levels have high *p,p*′‐DDE levels; however, further studies in temperate fruit orchards are needed to confirm these findings.

Comparisons of BSAFs and BMFs from earlier studies have been limited by foraging observation data, small sample sizes, and the use of wet or dry weight values instead of lipid equivalents. Harris et al. (2000) reported an average BSAF of 8.58 and BMF of 41.52 in Okanagan valley orchards for *p,p*′‐DDE, which was up to three times higher than those in Simcoe and Niagara where only ~40% of orchards had BSAFs >1, indicating slight regional differences in bioaccumulation. Vermeulen et al. (2010) found similar results for *p,p*′‐DDE in *L. rubellus* species with BSAFs ranging from 0.48 to 1.70 in grassland and woodland sites; however, samples were collected in only one sampling plot and earthworms were not depurated and contained soil, which likely resulted in an overestimate. Fremlin et al. (2020) estimated a trophic magnification factor of 7.8 for *p,p*′‐DDE in a Cooper's Hawk (*Accipiter cooperii*) food web (which included earthworms and American robins) across six sites in British Columbia and argued that the average biomagnification of *p,p*′‐DDE through these systems is higher than what has been measured in some aquatic food webs. Our average BSAFs for DDT‐r were all >1, confirming that earthworms are still capable of bioaccumulating aged DDT‐r through dietary exposure. Furthermore, although our orchards were within a ~5–10 km study area, the variation in our BMFs (i.e. 0.18–28) indicates that American robins undoubtedly have larger foraging ranges in orchards, which is likely contributing to the spatial variation in the earthworms they consume, but also in the proportion and species of worms, relative to other invertebrate prey ingested.

Several models have been proposed to explain the phenomenon of bioaccumulation and food web biomagnification. The fugacity principle has received increasing attention over the years, which contends that hydrophobic organic chemicals increase in concentration in an animal due to a thermodynamic gradient established in the gastrointestinal tract (Gobas et al., 1993). Indeed, thermodynamic studies in fish have shown that the fugacity of DDT‐r becomes elevated in the body due to the reduction of the volume of food during digestion and absorption, causing the non‐metabolizable chemical(s) to dissociate or be “squeezed” out of dietary lipids and passively diffuse through the mucosal membranes of the GIT where lipid co‐assimilation and other transport mechanisms can govern the internal distribution of DDT‐r in the body (Gobas et al., 1993, 1999). Our results conform to this theory because American robins can consume up to 14 feet of earthworms in a day (Sibley, 2020), which could result in higher dietary intake rates and absorption efficiencies to which metabolism and excretion are subsequently decreased (Hunt & Sacho, 1969a), thus, increasing the *p,p*′‐DDE concentration and BMF in eggs. By contrast, the low concentrations, fugacities, and BMFs of *p,p*′‐DDT in our study indicate that *p,p*′‐DDT does not have a propensity to biomagnify in American robins, likely owing to dietary biotransformation.

The bioaccumulation of *p,p*′‐DDE in American robin eggs and tissues is also influenced by various biological and toxicokinetic processes, such as maternal transfer. Due to their hydrophobic and lipophilic nature, *p,p*′‐DDE molecules could freely diffuse across the oocyte plasma membrane and/or bind to yolk precursors, such as very‐low‐density lipoproteins and vitellogenin, and be actively transported into the egg yolk at a higher concentration (Eng et al., 2012). Because yolk proteins are synthesized in the liver and therefore likely reflect reproductive investment among females, the observed increase in *p,p*′‐DDE fugacity in some of our robin eggs could be a function of reproductive status, age, and size (Arnot & Gobas, 2006) especially in female robins laying their first clutch who are likely to occupy a lower position relative to the steady state bioaccumulation potential of older robins. Similarly, egg‐laying order effects can result in varying degrees of dilution in the lipid‐normalized concentration of *p,p*′‐DDE relative to the maternal tissue (Drouillard & Norstrom, 2001). That may have some implications for the interpretation of DDT‐r data derived from sampling a single egg, especially in precocial birds with different breeding strategies, egg/clutch sizes, and egg‐laying periods (Braune et al., 2018). Whole‐body elimination rates and BMFs for *p,p*′‐DDE are also dependent on hepatic mono‐oxygenase activity, which is typically lower in birds compared to other vertebrates (Connell, 1990), as well as growth dilution, which can reduce a chemical's concentration and BMF due to the increase in the volume of tissue during different developmental stages (Gobas et al., 1999). The latter was partly observed by Harris et al. (2000) who found that American robin nestlings had lower *p,p*′‐DDE concentrations (mean 9.92 µg/g wet wt) than eggs (mean 26 µg/g) from the same nests; although, ontogenetic dietary shifts could have resulted in juveniles being fed fewer contaminated prey items (Drouillard et al., 2007) consequently diluting their body acquisitions.

American robins are considered a short‐distance migratory bird; however, western populations are known to exhibit some plasticity in the timing and route(s) of migration (Vanderhoff et al., 2020), which could bring a robin out of a steady state and increase the internal distribution of DDT‐r. Ulfstrand and Södergren (1972) simulated the stress encountered in migratory flights by subjecting caged robins to a period of starvation following an administration of 10.5 µg/g of DDT via diet and found that *p,p*′‐DDE concentrations were five‐fold greater in the body tissues of starved robins (which was attributed to the loss of fat reserves) compared to control birds. Alternatively, American robins that were contaminated at their wintering grounds in northern Maine between 1966 to 1973 lost nearly 90% of their ∑DDT burdens when females nested in uncontaminated forests (Knupp et al., 1976). Local banding data in the Okanagan valley confirms that over the past 20 years of standardized fall migration monitoring, American robins have been recaptured and the number of migrating flocks has increased with an average count of 400 robins per season between August and October (Vaseux Lake Bird Observatory, 2020). These findings are in agreement with other long‐term banding data in the Pacific Northwest, which show that American robins are travelling less than 100 km from their breeding areas and adapting a non‐migratory strategy possibly due to climate change (Brown & Miller, 2016). The 10 orchards in our study were well within a 100 km region, providing evidence that American robins in the Okanagan valley could have greater DDT‐r exposures over the annual cycle.

### Toxicological implications

The ecotoxicological effects of *p,p*′‐DDE in American robins has been studied under a range of field and laboratory conditions. Ten‐day old robin nestlings that were collected from DDT‐contaminated Okanagan valley orchards in 1997 weighed less and had shorter middle toes and tarsi compared to reference birds at 2, 5, and 9 months of age suggesting delayed development (Smith, 2004). In a follow up study, American robin nestlings from Okanagan valley orchards that were exposed to *p,p*′‐DDE in‐ovo (mean 55.8 µg/g wet wt) exhibited a 15% reduction in whole brain volume, a 13% reduction in relative forebrain size, and a 40% reduction in the size of the intercollicularis song nuclei with a concomitant increase in ∑DDT (Iwaniuk et al., 2006). The *p,p*′‐DDE metabolite is a potent androgen receptor antagonist that can inhibit testosterone production during embryonic and early post‐hatching phases, as well as disrupt Ca^2+^ uptake in cholinergic neurons, and decrease the expression of brain‐derived neurotrophic factor (Iwaniuk et al., 2006). Our concentrations, which were comparable to these studies, indicates that *p,p*′‐DDE can still reach levels known to affect the brain and song system in American robins.

Long‐term exposure to *p,p*′‐DDE in fruit orchards may also be associated with a decline in hatching success. For instance, Bishop et al. (2000) collected eggs of Eastern bluebirds (*Sialia sialis*), a closely related species to American robins, from southern Ontario apple orchards and observed a notable increase in unhatched eggs as *p,p*′‐DDE concentrations increased to 105 µg/g (wet wt) in bluebird eggs. The high *p,p*′‐DDE levels in our study suggest that these effects may be occurring in bluebirds and other migratory thrushes that nest/feed in Okanagan valley orchards. Elliott et al. (1994) also considered whether ongoing elevated concentrations of *p,p*′‐DDE in American robins (i.e. >50 µg/g whole body) would pose a health risk to local predators and birds of prey in the Okanagan valley. The diet of raptors such as Cooper's Hawks can include up to 85% of American robins during the breeding season (Cava et al., 2012). In addition, peregrine falcons (*Falco peregrinus*) were predicted to experience eggshell thinning from consuming 10% or more of American robins and other avian prey, such as European starlings (*S. vulgaris*), that contain *p,p*′‐DDE concentrations as low as 0.5 µg/g (wet wt; Elliott et al., 2005).

## CONCLUSION

Although several decades have passed since DDT was last applied in British Columbia, Canada, we found that American robins breeding in Okanagan valley fruit orchards are still being exposed to elevated concentrations of DDT‐r via a soil–earthworm–egg food chain. The highest ∑DDT soil concentrations were found in an apple orchard in Summerland (151 µg/g organic carbon‐lipid equivalent) and across 10 orchards, soil concentrations of *p,p*′‐DDE, *p,p*′‐DDT, and *p,p*′‐DDD averaged 16.1, 16.5, and 0.186 µg/g, respectively. *Lumbricidae* and *Aporrectodea* earthworms collected from these orchards also contained high *p,p*′‐DDE concentrations (species mean 96.5 µg/g lipid equiv.). Concentrations of DDT‐r were highly variable among individual robin eggs, with the highest concentration of *p,p′‐*DDE at 107 µg/g (wet wt) or 1,979 µg/g (lipid equiv.) confirming that contamination is still present at elevated levels similar to the 1990s. Biota‐soil‐accumulation factors and BMFs for DDT‐r across orchards were generally >1 and our fugacity analyses suggested that *p,p*′‐DDE is still thermodynamically biomagnifying in American robins 26 years after a previous study at the same sites/region. We conclude that the biomagnification of DDT‐r in American robin food chains is influenced by multiple factors, including historical application rates, patchiness in residual soil and earthworm contamination, and diet and foraging behaviour among individual females in fruit orchards. Concentrations of ∑DDT in our study exceeded published levels reported in other migratory birds nesting in temperate fruit orchards where reproductive effects were observed, and we recommend that future studies continue to monitor DDT‐r levels in free‐living terrestrial passerines and other wildlife that may be using orchard habitat.

## Supporting Information

The Supporting Information is availableon the Wiley Online Library at https://doi.org/10.1002/etc.5220.

## Conflict of Interests

The authors declare that they have no known competing financial interests or personal relationships that could have appeared to influence the work reported in the present study.

## Author Contributions Statement

R. Kesic: investigation, data curation, formal analysis, methodology, visualization, writing–original draft preparation, writing–review and editing. J.E. Elliott: conceptualization, project administration, supervision, resources, funding acquisition, writing–review and editing. K.M. Fremlin: investigation, data curation, methodology, writing–review and editing. L. Gauthier: resources, data curation, writing–review and editing. K.G. Drouillard: resources, data curation, writing–review and editing. C.A. Bishop: conceptualization, supervision, resources, writing–review and editing.

## Supporting information

This article includes online‐only Supporting Information.

Supporting information.

## Data Availability

Data, associated metadata, and calculation tools are available from the corresponding author (john.elliott@canada.ca). Data associated with this manuscript is also deposited in the Simon Fraser University Library Repository, Summit: http://summit.sfu.ca/item/21107.
