# Supplementary material for: Continuing Persistence and Biomagnification of DDT and Metabolites in Northern Temperate Fruit Orchard Avian Food Chains
Source: Environ Toxicol Chem. 2021 Nov 10;40(12):3379–91. doi: 10.1002/etc.5220 (PMC9299171; doi:10.1002/etc.5220)
Supplement: Supplementary file 1 — Supporting information. [file ETC-40-3379-s001.docx]

**Continuing persistence and biomagnification of DDT and metabolites in northern temperate fruit orchard avian food chains**

**Supplementary Information**

Pages: 8

**MATERIALS & METHODS**

***Soil analyses***

Soil samples were analysed at the Great Lakes Institute for Environmental Research (GLIER) in Windsor, ON as described by Drouillard et al. (2006). For each sample, approximately 20 g of soil was mixed with Na_2_SO_4_, spiked with either 50 µL of PCB-34 or 100 µL of PBDE-71 as recovery standards and then extracted with 150 mL of 50:50 acetone:hexane (ACE:HEX) using a Soxhlet extractor (Lab-Line 5000 Multi-Unit Extraction Heater, Lab Line Instruments Inc.). Each soil sample was rotary evaporated (Büchi Rotavapor RE 111) to 50 mL, mixed with 20% sodium chloride in 200 mL of Millipore grade water and extracted consecutively with 50, 25 and 25 mL of hexane in a separatory funnel. Soil extracts were rotary evaporated to a final amount of 2 mL and were subjected to a GPC and/or Florisil® clean-up phase before being transferred to 2 mL GC-MS vials. DDT-r, PCBs, PBDEs and other OCs in soil samples were analysed using similar GC-MS methods as described below.

***Chemical analyses for earthworms and American robin eggs***

American robin egg contents and earthworms were analyzed at the National Wildlife Research Centre (NWRC) in Ottawa, ON as described by Gauthier et al. (2007) and Gauthier et al. (2008). American robin eggs were briefly homogenized by whisking yolk and albumen together. Frozen earthworm tissue was thawed, cut into small pieces, and homogenized with a ball-mill (Retsch^TM^ MM400 Mixer Mill, Fisher Scientific). Approximately 0.25 to 3.0 g of each sample homogenate was accurately weighed, ground with diatomaceous earth (J.T. Baker, NJ, U.S.A.), and spiked with 25 μL of a ^13^C-labelled standard solution. Biota samples were extracted with a 50:50 dichloromethane:hexane (DCM:HEX) solvent mixture using an accelerated solvent extraction system (ASE, Dionex ASE 350, CA, USA). The bulk of lipids and other biogenic material were removed using gel permeation chromatography (GPC) (Gilson Inc., Middleton, WI, USA) and any residual lipids were removed with solid phase extraction (SPE). Each biota sample was then concentrated to 100 μL using nitrogen evaporation and added to 400 µL of iso-octane before instrumental analysis. DDT-r, polychlorinated biphenyls (PCBs) and other legacy organochlorines (OCs) in biota samples were analyzed using an Agilent 7890 gas chromatograph (Agilent Technologies, CA, USA) coupled to a triple-quadruple mass analyzer (Agilent 7000 MS) in electron impact ionization (MS-EI) mode. A ZB-5, Zebron multiresidue 30 m x 0.25 mm ID, 0.25 µm film thickness (Phenomenex, CA, USA) was selected because it provides optimal separation of chemical compounds, especially at higher concentrations. The injector was set to multi-mode, held at 70 °C, and ramped at 600 °C/min to 320 °C. The internal standards for the quantification of chemicals were all ^13^C-labelled (Cambridge Isotope Laboratories, MA, USA).

Low molecular weight polybrominated diphenyl ethers (PBDEs) were analysed in biota samples using an Agilent 7890 gas chromatography (Agilent Technologies, CA, USA) coupled to a single quadruple mass analyzer (Agilent 5973 MS) in MS-EI mode. High molecular weight PBDEs (i.e., BDE-197, -196, -206, -207, -209) were analysed using an Agilent 6890 gas chromatograph coupled to a GCT high resolution time-of-flight (TOF) mass spectrometer (Micromass, Manchester, UK) in EI mode. Internal standards for the quantification of PBDEs were all ^13^C-labelled.

***Stable isotope analyses***

American robin eggs and earthworm samples were analyzed for naturally-occurring stable isotopes of carbon (δ^13^C) and nitrogen (δ^15^N). Samples were briefly freeze-dried, ground up with a polytron and ball-mill and weighed (~1 mg) into 6 mm tin capsules. Samples were combusted at 1800 °C in a Vario EL Cube elemental analyzer (Elementar, Germany) interfaced to a Delta Advantage isotope ratio mass spectrometer (IRMS; Conflo IV, Thermo Scientific, Germany). The internal standards used for δ^13^C (in ‰) included: C-51 Nicotiamide (0.07, -22.95), C-52 mix of ammonium sulphate and sucrose (16.58, -11.94), C-54 caffeine (-16.61, -34.46), and blind standard C-55 glutamic acid (-3.98, -28.53). These standards cover the natural range, and the data is reported in Delta notation δ, with units in per mil (‰) and are expressed as:

$$\delta X= \frac{{(R}_{sample}-R_{standard})}{R_{standard}\times1000}$$

where δX is ^15^N or ^13^C, and R is the ratio of the abundance of the heavy to the light isotope in the biota sample. All δ15N is reported as ‰ vs. AIR and normalized to internal standards calibrated to International standards IAEA-N1 (+0.4‰), IAEA-N2 (+20.3‰), USGS-40 (-4.52‰) and USGS-41 (47.57‰). All δ13C is reported as ‰ vs. V-PDB and normalized to internal standards calibrated to International standards IAEA-CH-6 (-10.4‰), NBS- 22 (-29.91‰), USGS-40 (-26.24‰) and USGS-41 (37.76‰).

***Quality control and assurance***

A sample field blank was collected during soil, earthworm, and American robin egg sampling to monitor for possible background interference and contamination from handling. Field blank collections consisted of opening an empty chemically rinsed jar during each sampling activity and closing it upon completion of sorting. Method procedural blanks were also processed with each extraction batch to monitor for background interference. All chemical concentrations were blank corrected. Analytical accuracy and precision of contaminant concentration data were evaluated by running an aliquot of a Certified Standard Reference Material (SRM; NIST 1947 Lake Michigan Fish Tissue), as well as duplicates of randomly selected egg or earthworm samples. For each contaminant, the method detection limit (MDL) was defined as the minimum measured concentration of analyte producing a peak with a signal to noise ratio of 3. The MDLs for American robin eggs and earthworm analyses for *p,p’-*DDE, *p,p’*-DDT, and *p,p’*-DDD were 0.0008 µg/g, 0.0012 µg/g and 0.0006 µg/g (ww), respectively. The MDLs for soil analyses for *p,p’*-DDE, *p,p*’-DDT and *p,p*’-DDD were 0.000024, 0.000061, and 0.000031 µg/g dry weight (respectively).

***Lipid normalization and fugacity***

Soil samples were expressed as organic carbon-lipid equivalent fractions (C*_OC-equiv._*; µg/g of OC-equivalent) and were based on the following equation:

$$C_{organic carbon-lipid equivalent}= \frac{C_{dry}}{\mathrm{OC}_{dry}\left( 0.35 \right)}$$

in which C_dry_ is the concentration of the soil sample in dry weight (µg/g), OC_dry_ is the fraction of the total organic carbon in the soil sample (g of OC/g of dry weight) and the 0.35 is a proportionality constant that assumes that organic carbon has 35% of the sorptive capacity relative to that of octanol (L/kg OC), which is consistent with near equilibrium (Mackay, 2004; Kelly et al., 2007; deBruyn and Gobas, 2009).

Lipid contents were measured in American robin eggs and earthworm samples using a gravimetric method. DDT-r concentrations in robin eggs and earthworm samples were expressed as lipid equivalent fractions (C*_lipid equiv_*.; µg/g lipid equivalent) based on the following equation:

$$C_{lipid-equivalent}= \frac{C_{wet}}{L_{wet}+ NLOM\left( 0.05 \right)}$$

in which L is the lipid fraction of the sampled tissue (g of lipid/g of wet tissue) and NLOM is the non-lipid organic matter, estimated as the dry weight subtracted from the lipid content of the sample. The constant 0.05 is based on non-lipid organic matter exhibiting 5% of the sorptive capacity of lipids (deBruyn and Gobas, 2009).

DDT-r activities in soil, earthworms, and American robin eggs were also expressed as chemical fugacities to facilitate the comparison of chemical equilibrium between DDT-r in abiotic (i.e., soil) and biotic (i.e., earthworms and robin eggs) media. Fugacity (*f* = *C*/*Z*; in units of Pa or nPa) was determined from the measured DDT-r concentration in each medium (*C;* mol/m^3^) converted from the weight concentration (µg/kg dry weight for soil or µg/kg wet weight for biota) and the fugacity capacity (*Z;* mol/Pa⋅m^3^) of DDT-r in each medium (Mackay, 2004). DDT-r concentrations (*C*) in each environmental media were calculated as follows:

$$C_{\left( \frac{mol}{m3} \right)}=C_{x\left( \frac{\mu g}{kg} \right)}\times\frac{D_{X}}{{MW\times10}^{6}}$$

where C_X_ is the DDT-r concentration in the media (µg/kg), D_X_ is the bulk density of the media (assumed to be 1300 kg dry weight per m^3^ for orchard soils) or biota (1000 kg wet weight/m^3^) and MW is the molecular weight (g/mol) of the chemical. The fugacity capacity for soil was calculated based on the following equation described by Mackay and Paterson (1981):

$$Z_{Soil}=\frac{K_{soil,wat}\times D_{S}}{H}$$

where K_soil,wat_ is the soil/water partition coefficient (L/kg), D_S_ is the bulk density of soil (assumed to be 1.3 kg dry weight/L) and H is the Henry’s Law Constant for the chemical (Pa·m^3^/mol). However, K_soil,wat_ is typically not available but can be estimated from K_OW_ such that:

$$K_{soil,wat}\approx f_{OC}\times K_{OC}\approx f_{OC}\times0.35\times K_{OW}$$

where f_OC_ is the fraction of organic carbon in soil (kg OC/kg dry weight), K_OC_ is the organic carbon/water partition coefficient (L water/kg OC); 0.35 is a proportionality coefficient (L oct/kg OC) relating the chemical partition capacity of organic carbon relative to octanol and K_OW_ is the octanol/water partition coefficient (L wat/L oct; unitless).

The fugacity capacity in biota for each DDT-r compound was determined as:

$$Z_{biota}=\frac{K_{biota,wat}\times D_{B}}{H}$$

where K_biota/wat_ is the organism/water partition capacity, D_B_ is the density of biota (assumed to be 1 kg wet weight/L) and H is the Henry’s Law Constant for the chemical (Pa·m^3^/mol). Similar to the soil/water partition coefficient, K_biota,wat_ can be estimated from K_OW_ by accounting for the lipid and NLOM content of the animal:

$$K_{biota,wat}\approx{(f}_{lipid}\times1)+(f_{NLOM}0.05)\times K_{OW}$$

Where f_lipid_ and f_NLOM_ are the fractions of lipid (kg lipid/kg wet weight) and non-lipid organic matter (kg NLOM/kg wet weight); the constants 1 and 0.05 refer to the relative partition capacity of lipids (L oct/kg lipid) and NLOM (L oct/kg NLOM), respectively.

**REFERENCES**

deBruyn AM, Gobas FA. 2009. The Sorptive Capacity of Animal Protein. *Environmental Toxicology and Chemistry* 26:1803-1808.

Drouillard KG, Tomczak M, Reitsma S, Haffner GD. 2006. A River-wide Survey of Polychlorinated Biphenyls (PCBs), Polycylic Aromatic Hydrocarbons (PAHs), and Selected Organochlorine Pesticide Residues in Sediments of the Detroit River—1999. *Journal of Great Lakes Research* 32:209-226.

Gauthier LT, Hebert CE, Chip Weseloh, DV, Letcher, RJ. 2007. Current-use flame retardants in the eggs of herring gulls (*Larus argentatus*) from the Laurentian Great Lakes. *Environmental Science and Technology* 41:4561-4567.

Gauthier LT, Hebert CE, Chip Weseloh, DV, Letcher, RJ. 2008. Dramatic changes in the temporal trends of polybrominated diphenyl ethers (PBDEs) in herring gull eggs from the Laurentian Great Lakes: 1982–2006. *Environmental Science and Technology* 42:1524-1530.

Kelly BC, Ikonomou MG, Blair JD, Morin AE, Gobas FA. 2007. Food web-specific biomagnification of persistent organic pollutants. *Science* 317:236-239.

Mackay D, Paterson S. 1981. Calculating fugacity. *Environmental Science and Technology* 15:1006-1014.

Mackay D. 2004. Finding fugacity feasible, fruitful, and fun. *Environmental Toxicology and Chemistry* 23:2282-2289.
